# Diffusion anisotropy as an imaging biomarker of directional progression in temporal glioblastoma

**DOI:** 10.1007/s11060-026-05851-6

**Published:** 2026-11-12

**Authors:** Jawad Fares, Yonghao Li, Yizhou Wan, Stephen J. Price

**Affiliations:** 1 Cambridge Brain Tumour Imaging Laboratory, Department of Clinical Neurosciences, University of Cambridge, Cambridge, UK; 2 Academic Neurosurgery Division, Department of Clinical Neurosciences, University of Cambridge, Box 167 Cambridge Biomedical Campus, Cambridge, CB2 0QQ UK; 3 Department of Neurological Surgery, Feinberg School of Medicine, Northwestern University, Chicago, IL USA

**Keywords:** Glioblastoma, Diffusion tensor imaging, White matter tracts, Tumor invasion, Surgical planning

## Abstract

**Purpose:**

Glioblastoma frequently progresses beyond surgical margins, yet whether preoperative imaging can predict its direction remains unclear. Given the anatomical characteristics of the temporal lobe and the potential role of anterior temporal lobectomy, we evaluated FLAIR, isotropic diffusion (DTI-p), and diffusion anisotropy (DTI-q) as imaging biomarkers of directional progression after complete resection.

**Materials and methods:**

We retrospectively analyzed a prospectively recruited multicenter cohort of patients with newly diagnosed IDH-wildtype glioblastoma confined to the temporal lobe who underwent complete resection of contrast-enhancing tumor and had preoperative diffusion tensor imaging and follow-up MRI demonstrating progression. Progression was visually classified relative to the resection cavity. Direction-specific accuracy, sensitivity, and specificity were calculated, with paired comparisons using exact McNemar tests and Holm correction across all comparisons. Times to progression and death were summarized descriptively.

**Results:**

Thirty-two patients met inclusion criteria. DTI-q demonstrated the highest predictive accuracy across all progression directions (median, 75%), compared with DTI-p (median, 50%) and FLAIR (median, 44%). DTI-q significantly outperformed FLAIR and DTI-p for lateral, superior, and inferior progression after Holm correction. Combined imaging models did not improve predictive performance over DTI-q alone.

**Conclusion:**

Preoperative DTI-q predicted directional progression after complete resection more accurately than FLAIR or DTI-p, supporting DTI-q as a potential imaging biomarker for spatially informed surgical planning.

## Introduction

Glioblastoma remains the most aggressive primary brain tumor, characterized by diffuse infiltration and near-universal recurrence despite maximal therapy [1, 2]. Even after complete resection of contrast-enhancing tumor followed by chemoradiotherapy, most recurrences arise locally at or near the resection margin, reflecting microscopic disease extending beyond radiographically visible tumor boundaries [3–5].

In temporal lobe glioblastoma, this challenge is particularly relevant given the complex anatomy and the presence of dense white matter pathways that may facilitate preferential routes of tumor spread [6, 7]. Emerging surgical strategies, including supramaximal resections such as anterior temporal lobectomy, have been proposed to address this limitation by targeting infiltrative tumor beyond the enhancing core [8–10]. However, the effectiveness of such approaches depends fundamentally on understanding where tumors are most likely to recur and whether these patterns follow predictable anatomical trajectories.

Our prior single-center retrospective study demonstrated nonrandom progression patterns in temporal lobe glioblastoma but limited accuracy of FLAIR for predicting progression direction [11]. Whether diffusion-based biomarkers can better anticipate these trajectories remains unclear. Diffusion tensor imaging (DTI) captures white matter microstructure, including directional diffusion reflecting fiber architecture [12–14]. Prior multicenter work from the PRaM-GBM study demonstrated spatial associations between preoperative diffusion anisotropy (DTI-q) abnormalities and subsequent progression using voxel-based analysis [15, 16]. However, whether preoperative imaging can predict the anatomical direction of progression relative to the surgical cavity, and whether DTI-q outperforms FLAIR or diffusion isotropy (DTI-p) for this purpose, remains unknown. We therefore characterized progression after complete resection in a multicenter cohort and compared FLAIR, isotropic diffusion (DTI-p), and diffusion anisotropy (DTI-q) as predictors of direction-specific progression.

## Methods

### Patient cohort and eligibility

This study involved retrospective analysis of a prospectively assembled multicenter cohort of adults with newly diagnosed IDH-wildtype temporal lobe glioblastoma. The cohort was independent of the previously reported single-center temporal cohort [11], with no patient overlap. Participating institutions included Cambridge University Hospitals NHS Foundation Trust (Addenbrooke Hospital), James Cook University Hospital, Leeds Teaching Hospitals NHS Trust, Nottingham University Hospitals NHS Trust, and The Walton Centre NHS Foundation Trust. Inclusion required a primary tumor confined to the temporal lobe, preoperative MRI including DTI, immediate postoperative MRI sufficient to assess resection, complete resection of contrast-enhancing tumor, and follow-up MRI demonstrating radiographic progression. The study had ethical approval, and patients provided informed written consent. Temporal-lobe confinement was assessed on anatomical MRI by an expert neurosurgeon. Anatomical references included the temporal pole, Sylvian fissure, superior temporal gyrus, temporal horn, and mesial temporal structures (uncus, amygdala, and hippocampus) [17].

Patients were excluded for tumor extension beyond the temporal lobe, inadequate DTI or longitudinal imaging, prior cranial radiotherapy before the index surgery, re-resection before documented first progression, or incomplete resection.

Complete resection was defined as no residual contrast enhancement on immediate postoperative T1-weighted MRI. Intraoperative adjuncts were used at the treating neurosurgeon’s discretion.

Clinical and demographic data included age, sex, tumor laterality, periventricular involvement, KPS, and MGMT promoter methylation status.

### MRI protocol and DTI processing

Preoperative MRI included T1-weighted imaging with and without contrast, T2-weighted FLAIR, and DTI. Imaging acquisition followed the previously published protocol [18]. Preoperative imaging was performed on a 3-T Siemens Magnetom system with a 12-channel head coil. DTI used a single-shot echo-planar sequence (TR/TE, 8300/98 ms; flip angle, 90 degrees; field of view, 192 × 192 mm; 63 slices without a gap; 2-mm isotropic voxels), with b-values of 0, 350, 650, 1000, 1300, and 1600 s/mm2 sampled in 13 directions. DTI was obtained before contrast administration. Imaging datasets were reviewed for quality, and studies with significant artifacts were excluded. Preoperative MRI was obtained within seven days before surgery. Postoperative MRI was obtained 24–48 h after surgery, and no later than 72 h.

DTI preprocessing was performed using FMRIB Software Library (version 5.0), including eddy current correction and tensor fitting [19]. Diffusion tensors were decomposed into isotropic (DTI-p) and anisotropic (DTI-q) components using the validated p: q decomposition method [20, 21]. In this framework, DTI-p represents the isotropic diffusion component and DTI-q the anisotropic component [13]. 

Parametric DTI maps and FLAIR images were linearly coregistered to the preoperative contrast-enhanced T1-weighted volume using FLIRT. Postoperative and follow-up MRI were aligned to this reference using the previously described semiautomatic workflow [18]. Brain-extracted images were manually corrected, and FLIRT registration using lesion or resection-cavity, ventricular, and external brain masks provided the initial transformation for subsequent nonlinear registration. Progression was assessed within the cavity-anchored directional framework described for frontal glioblastoma [16]. Registration accuracy was not quantitatively validated in this cohort, and residual postoperative deformation may introduce spatial uncertainty.

### Biomarker segmentation and directional assignment

Preoperative FLAIR, DTI-p, and DTI-q abnormalities were manually segmented relative to contralateral normal-appearing white matter, without fixed intensity thresholds. Masks were independently reviewed by two neurosurgeons and finalized by consensus before directional assignment.

Each biomarker was considered positive along six anatomical axes (anterior, posterior, medial, lateral, superior, inferior) when abnormal signal extended beyond the enhancing tumor boundary, without minimum distance or volume thresholds. Directions were non-mutually exclusive, and assessments were performed blinded to progression and survival outcomes.

Progression was defined as new or enlarging contrast enhancement on follow-up MRI. When treatment-related changes or pseudoprogression were suspected, subsequent imaging was reviewed to establish persistent or progressive enhancement. Only radiographically confirmed progression was included in the directional analysis.

First progression was visually classified relative to the postoperative cavity as anterior, posterior, medial, lateral, superior, inferior, or distant, with non-mutually exclusive assignments. An expert neurosurgeon assessed progression directions, with consensus review as described above and blinding to preoperative biomarkers. In this study, “distant” denoted invasion beyond the temporal lobe (extratemporal progression). For each patient, the six anatomical directions were independently classified as positive or negative for each preoperative imaging biomarker and for subsequent progression. Directions were non-mutually exclusive, and all 32 patients contributed to each direction-specific comparison.

### Surgical feasibility assessment

The anatomical compatibility of observed progression patterns with anterior temporal lobectomy was assessed retrospectively using preoperative and follow-up imaging. Assessment considered the location and extent of progression relative to the anterior temporal lobe, the contrast-enhancing tumor, and relevant neurosurgical anatomical landmarks, including proximity to critical white matter pathways. The anticipated extent of resection was evaluated within the study’s predefined anterior temporal lobectomy boundaries [17].

### Outcomes and statistical analysis

Progression-free survival (PFS) was defined as the interval from surgery to radiographic progression or death, whichever occurred first. Overall survival (OS) was defined as the interval from surgery to death from any cause, with patients remaining alive censored at their last documented follow-up.

Continuous variables were summarized as medians with interquartile ranges (IQRs), using linear-interpolation quartiles, and categorical variables as frequencies and percentages. Time intervals were converted from days to months using 30.4375 days per month. Progression directions and imaging biomarkers were analyzed as non-mutually exclusive binary variables. The number of positive directions per patient was summarized for each imaging modality and observed progression. Pairwise overlap between biomarker direction sets was quantified using the Jaccard index, defined as the intersection divided by the union of positive directions within each patient.

For each anatomical direction, the predictive performance of FLAIR, DTI-p, and DTI-q was evaluated using observed progression as the reference. Accuracy was defined as (TP + TN)/(TP + FP+FN + TN), sensitivity as TP/(TP + FN), and specificity as TN/(TN + FP), where TP, TN, FP, and FN denote true positives, true negatives, false positives, and false negatives, respectively. All 32 patients contributed to each direction-specific analysis. Imaging biomarkers were compared using exact two-sided McNemar tests applied to paired correct/incorrect classifications, with one paired observation per patient per direction.

The exploratory analysis comprised all three pairwise comparisons among FLAIR, DTI-p, and DTI-q across six anatomical directions (18 comparisons). Holm correction was applied across these comparisons to account for multiple testing. Pooled performance across 192 patient-direction observations was also evaluated, with 95% confidence intervals estimated using 50,000 whole-patient bootstrap resamples to account for within-patient clustering. Combined imaging biomarkers were evaluated using logical OR rules, whereby a direction was classified as positive if any constituent biomarker was positive. Comparisons involving combined biomarkers were considered secondary exploratory analyses.

Associations between clinical characteristics (age, KPS, sex, tumor laterality, periventricular involvement, and MGMT promoter methylation status) and the seven progression categories were explored using two-sided Fisher exact tests for categorical variables and Mann–Whitney U tests for continuous variables. Holm correction was applied across these 42 comparisons. All statistical tests were two-sided, with statistical significance defined as an adjusted p-value < 0.05. Statistical analyses were performed using Python (version 3.14; Python Software Foundation), with NumPy, SciPy, and statsmodels. Custom Python scripts were used for paired comparisons, multiple-testing correction, and patient-level bootstrap resampling.

## Results

### Patient characteristics

A total of 32 patients with temporal lobe glioblastoma met inclusion criteria, all with complete resection of contrast-enhancing tumor. Seventeen patients (53.1%) were aged 50–64 years, 25 (78.1%) were male, and 18 (56.2%) had left-sided tumors. Periventricular involvement was present in 12 (37.5%), and MGMT promoter methylation in 15 (46.9%). Median KPS was 90 [IQR, 90–100]. Median PFS was 9.1 months [7.3–13.9]. Median OS was 14.6 months [10.5–18.1] **(**Table 1**).**

**Table 1:** Demographic and baseline characteristics of patients with temporal lobe glioblastoma (*N* = 32)

Patient characteristic	*N* (%)
**Age**	
< 50 years	5 (15.6)
50–64 years	17 (53.1)
≥ 65 years	10 (31.2)
**Sex**	
Female	7 (21.9)
Male	25 (78.1)
**Tumor side**	
Right	14 (43.8)
Left	18 (56.2)
**Periventricular involvement**	
Yes	12 (37.5)
No	20 (62.5)
**Extent of resection of contrast-enhancing tumor**	
Complete resection	32 (100)
**MGMT promoter methylation**	
Unmethylated	16 (50.0)
Methylated	15 (46.9)
Unknown	1 (3.1)
**KPS**	
Mean ± SD	92.8 ± 8.5
Median [IQR]	90 [90–100]
**PFS**,** median [IQR] (months)**	9.1 [7.3–13.9]
**OS**,** median [IQR] (months)**	14.6 [10.5–18.1]

No clinical or demographic characteristic was significantly associated with progression direction after correction for multiple comparisons.

### Directional patterns of tumor progression

Posterior progression occurred in 21 patients (65.6%), medial in 18 (56.2%), anterior in 12 (37.5%), superior in six (18.8%), lateral in four (12.5%), and inferior in one (3.1%). Distant progression occurred in six patients (18.8%). Categories were nonexclusive **(**Table 2**).** The median number of anatomical progression directions was 2 [IQR, 1–2.25], with a range of 0–6.

**Table 2:** Progression patterns and outcome times in temporal lobe glioblastoma (*N* = 32)

Progression Pattern	*n* (%)	Median PFS (months)	PFS IQR (months)	Median OS (months)	OS IQR (months)
Anterior	12 (37.5)	8.5	6.0–13.6	14.8	13.0–19.4
Posterior	21 (65.6)	9.1	7.7–14.6	14.4	10.5–17.7
Medial	18 (56.2)	9.0	7.7–14.5	14.6	11.3–20.7
Lateral	4 (12.5)	7.1	6.4–7.7	14.8	12.8–16.7
Superior	6 (18.8)	10.4	7.7–15.5	21.5	14.6–26.1
Inferior	1 (3.1)	7.9	-	14.6	-
Distant	6 (18.8)	11.3	6.2–13.3	14.6	12.0–23.1

### Predictive performance of imaging biomarkers

DTI-q had the highest accuracy point estimate in each direction, with a median across six directions of 0.750 (range, 0.594–0.969), compared with 0.500 for DTI-p and 0.438 for FLAIR **(**Table 3**)**. Sensitivity and specificity did not consistently favor DTI-q. For inferior progression, DTI-q achieved 96.9% accuracy and 100% specificity but did not identify the single observed case, yielding 0% sensitivity.

**Table 3:** Direction-specific predictive performance (*N* = 32)

Direction	Biomarker	Accuracy	Sensitivity	Specificity
Anterior	FLAIR	0.438	1.000	0.100
	DTI-p	0.469	0.917	0.200
	**DTI-q**	**0.594**	**0.750**	**0.500**
Posterior	FLAIR	0.625	0.857	0.182
	DTI-p	0.562	0.762	0.182
	**DTI-q**	**0.688**	**0.905**	**0.273**
Medial	FLAIR	0.594	0.722	0.429
	DTI-p	0.625	0.833	0.357
	**DTI-q**	**0.750**	**0.778**	**0.714**
Lateral	FLAIR	0.375	0.500	0.357
	DTI-p	0.375	0.500	0.357
	**DTI-q**	**0.750**	**0.500**	**0.786**
Superior	FLAIR	0.438	0.833	0.346
	DTI-p	0.438	0.667	0.385
	**DTI-q**	**0.844**	**0.500**	**0.923**
Inferior	FLAIR	0.406	0.000	0.419
	DTI-p	0.531	0.000	0.548
	**DTI-q**	**0.969**	**0.000**	**1.000**

The median number of preoperative positive directions per patient was 4.5 [IQR, 3–5] for FLAIR, 4.5 [3–5] for DTI-p, and 2 [2–3] for DTI-q. Mean within-patient Jaccard overlap was 0.835 for FLAIR/DTI-p, 0.473 for FLAIR/DTI-q, and 0.482 for DTI-p/DTI-q. Pooled directional accuracy was highest for DTI-q (76.6%), compared with DTI-p (50.0%) and FLAIR (47.9%). Combining imaging biomarkers did not improve accuracy over DTI-q alone, with pooled accuracies ranging from 46.9% to 51.0%.

**Fig. 1:**
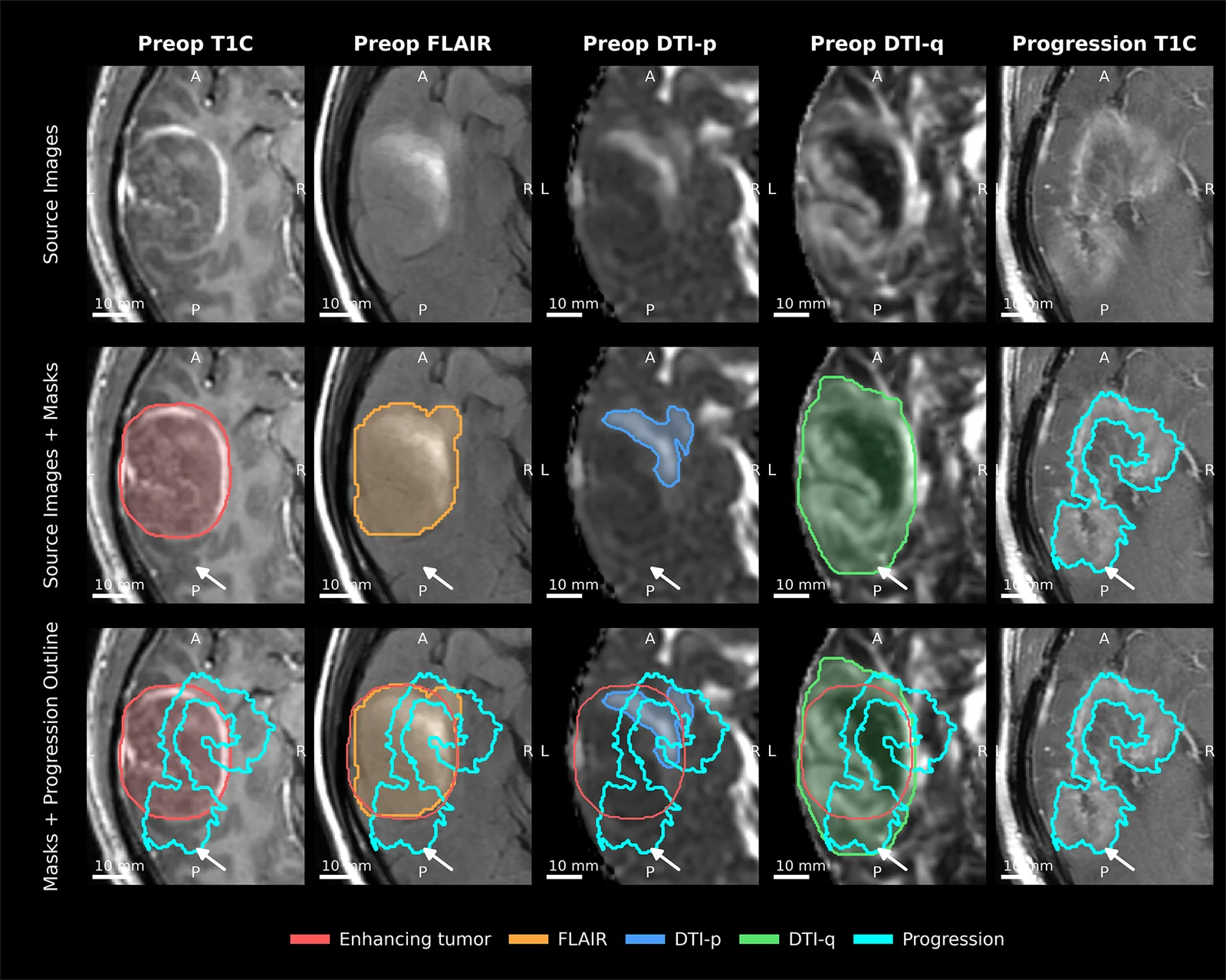
Preoperative imaging biomarkers and subsequent posterior progression in temporal lobe glioblastoma. Columns show preoperative T1C, FLAIR, DTI-p, DTI-q, and follow-up progression T1C. Rows display source images (top), individual segmentation masks (middle), and masks overlaid with subsequent progression (bottom). White arrows highlight posterior progression extending beyond FLAIR and DTI-p abnormalities but within the DTI-q abnormality. All images are coregistered to the preoperative reference space

**Fig. 2:**
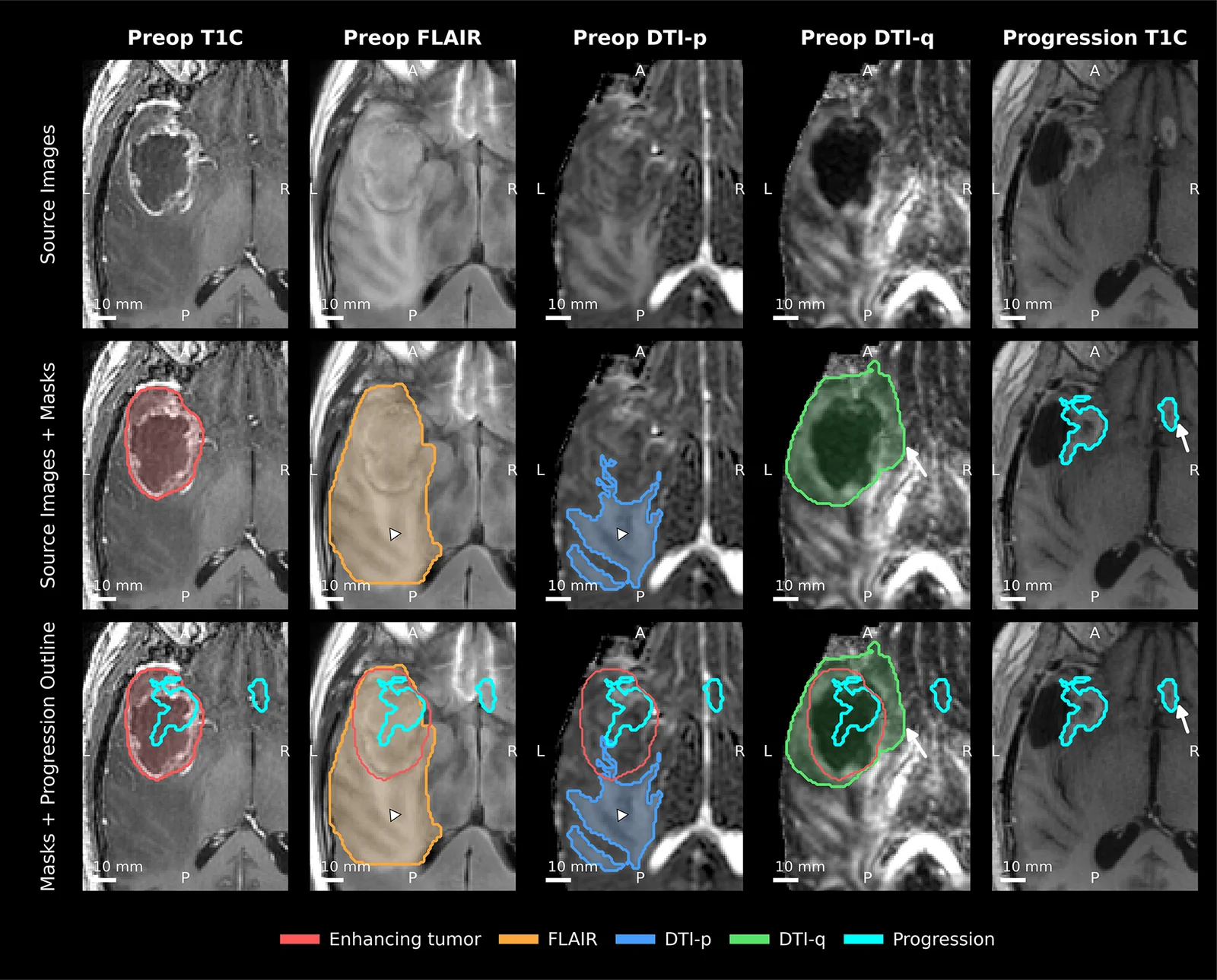
Preoperative imaging biomarkers and subsequent medial progression in temporal lobe glioblastoma. Columns and rows follow Fig. 1. White arrows highlight medial extension of the DTI-q abnormality and subsequent medial progression, while white arrowheads indicate posterior FLAIR and DTI-p abnormalities without corresponding posterior progression. All images are coregistered to the preoperative reference space

Two selected patients illustrate posterior prediction and medial prediction with posterior overextension by FLAIR and DTI-p **(**Figs. 1 and 2**)**.

### Paired comparisons of predictive accuracy

Exact paired tests showed higher DTI-q accuracy than both FLAIR and DTI-p for lateral, superior, and inferior directions after Holm correction **(**Table 4**)**. Anterior, posterior, and medial differences were not significant.

**Table 4:** Exact paired accuracy comparisons of DTI-q with FLAIR and DTI-p

Direction	DTI-q versus FLAIR					DTI-q versus DTI-*p*				
	Accuracy difference	Correct only with FLAIR (*n*)	Correct only with DTI-q (*n*)	Raw *p* value	Holm adjusted *p* value	Accuracy difference	Correct only with DTI-*p* (*n*)	Correct only with DTI-q (*n*)	Raw *p* value	Holm adjusted *p* value
Anterior	+ 0.156	3	8	0.2266	1.0000	+ 0.125	4	8	0.3877	1.0000
Posterior	+ 0.062	3	5	0.7266	1.0000	+ 0.125	2	6	0.2891	1.0000
Medial	+ 0.156	3	8	0.2266	1.0000	+ 0.125	3	7	0.3438	1.0000
Lateral	+ 0.375	1	13	0.0018	**0.0275**	+ 0.375	1	13	0.0018	**0.0275**
Superior	+ 0.406	2	15	0.0023	**0.0305**	+ 0.406	1	14	0.0010	**0.0156**
Inferior	+ 0.562	0	18	< 0.0001	**0.0001**	+ 0.438	0	14	0.0001	**0.0021**

*N* = 32 paired patients. Accuracy difference = DTI-q minus comparator. P-values: exact two-sided McNemar tests; Holm correction across 18 comparisons. Bold indicates adjusted *p* < 0.05

### Surgical implications

In 14 of 32 patients (43.8%), the observed progression pattern was anatomically compatible with an anterior temporal lobectomy, highlighting the potential relevance of directional progression mapping to surgical planning.

## Discussion

In this exploratory cohort of temporal lobe glioblastoma, posterior and medial progression predominated. DTI-q demonstrated higher classification accuracy than FLAIR and DTI-p across all six anatomical directions, with statistically significant advantages for lateral, superior, and inferior progression after correction for multiple comparisons. The rarity of inferior progression warrants cautious interpretation of its classification accuracy, while associations with survival remain uncertain. Retrospective anatomical assessment identified 14 patients (43.8%) with progression patterns compatible with anterior temporal lobectomy, highlighting the potential clinical relevance of directional imaging for surgical planning.

Glioblastoma invasion is spatially organized by brain architecture rather than uniform radial expansion, with white matter pathways providing preferential routes of spread [6, 12, 22–27]. The predominance of posterior and medial progression in our cohort is consistent with this framework and suggests that anatomical connectivity continues to shape progression after complete resection of contrast enhancement. Importantly, our findings extend this established biological concept by showing that progression direction can be anticipated relative to the surgical cavity.

The higher directional accuracy of DTI-q may reflect differences in the tissue characteristics captured by each imaging modality. FLAIR abnormalities encompass a broad spectrum of changes, including edema and infiltrative tumor [28], whereas DTI-q characterizes the anisotropic component of diffusion associated with white matter microstructure [14, 29]. In our cohort, DTI-q identified fewer abnormal directions per patient than FLAIR or DTI-p, potentially improving spatial discrimination by reducing false-positive directional predictions. However, this advantage varied across anatomical directions and was not accompanied by uniformly higher sensitivity.

Other imaging approaches, including perfusion, diffusion, and radiomics, have been investigated for identifying infiltrative tumor or predicting recurrence [30, 31]. Unlike these predominantly scalar or regional measures, DTI-q directly captures anisotropic white matter organization, providing a mechanistically plausible basis for direction-specific prediction.

Our prior single-center retrospective study demonstrated nonrandom temporal lobe progression and limited accuracy of FLAIR for predicting its direction [11]. The present prospectively recruited multicenter cohort was entirely independent, with no patient overlap. Notably, the predominance of posterior and medial progression in the present cohort differs from the anterior predominance observed previously, suggesting intercohort heterogeneity in temporal lobe progression patterns. This variability further supports the need for patient-specific imaging biomarkers rather than reliance on anatomical location alone to anticipate progression direction. Unlike the prior PRaM-GBM voxel-based analysis [15], the present study evaluates anatomical directionality relative to the surgical cavity and directly compares FLAIR, DTI-p, and DTI-q as direction-specific biomarkers.

These findings have potential implications for surgical planning in temporal lobe glioblastoma. While extended resections have traditionally emphasized the removal of non-enhancing abnormalities beyond the contrast-enhancing tumor [9, 10], directional imaging may offer an additional framework for identifying anatomically relevant regions at risk of progression. The observation that 43.8% of patients exhibited progression patterns compatible with anterior temporal lobectomy supports further investigation of individualized resection strategies informed by preoperative imaging. Whether such approaches translate into improved clinical outcomes remains to be determined.

This study has several limitations. Although patients were prospectively recruited, the analysis was retrospective and included a relatively small cohort restricted to patients with documented progression. The low frequency of progression in certain directions, particularly inferior progression, limits the precision and clinical interpretation of direction-specific performance estimates. Biomarker segmentation and progression classification relied on qualitative assessments and consensus review without formal quantification of interobserver agreement. Furthermore, anatomical constraints and resection geometry may influence directional classification, while potential interinstitutional imaging variability and residual registration uncertainty could affect spatial accuracy. The direction-based approach provides a clinically interpretable assessment of progression but does not establish voxel-level correspondence between preoperative imaging abnormalities and subsequent recurrence. Finally, the findings require validation in larger independent cohorts, and the potential benefit of imaging-guided extended resection remains to be established prospectively.

## Conclusions

Preoperative DTI-q demonstrated higher directional classification accuracy than FLAIR and DTI-p across all six anatomical directions in temporal lobe glioblastoma. These findings support further investigation of diffusion anisotropy as an imaging biomarker for spatially informed surgical planning.

## Data Availability

The Python analysis scripts and supporting documentation used to generate the reported results are available from the corresponding author upon reasonable request. De-identified patient-level data may also be made available, subject to institutional approval and applicable ethical and data-sharing requirements.

## Declarations

### AI declaration statement

During the preparation of this work the authors used Grammarly AI to improve the readability and language of the manuscript. After using this tool/service, the authors reviewed and edited the content as needed and take full responsibility for the content of the published article.
